# Systemic inflammation and sympathetic activation in gestational diabetes mellitus with obstructive sleep apnea

**DOI:** 10.1186/s12890-022-01888-1

**Published:** 2022-03-18

**Authors:** Oleksandr Serednytskyy, Alberto Alonso-Fernández, Caterina Ribot, Andrea Herranz, Ainhoa Álvarez, Andrés Sánchez, Paula Rodríguez, Ana V. Gil, Carla Pía, José P. Cubero, María Barceló, María Cerdà, Mercedes Codina, Mónica D. Peña, Antònia Barceló, Amanda Iglesias, Daniel Morell-Garcia, José A. Peña, María P. Giménez, María C. Piñas, Francisco García-Río

**Affiliations:** 1 Institut d’Investigació Sanitària Illes Balears (IdISBa), Palma, Balearic Islands Spain; 2 Servicio de Neumología, Hospital Universitari Son Espases, Carretera de Valldemosa 79, 07010 Palma de Mallorca, Balearic Islands Spain; 3 CIBER Enfermedades Respiratorias, Madrid, Spain; 4 Unidad del Sueño, Hospital Universitari de Araba, Vitoria-Gasteiz, Ávala Spain; 5 Instituto de Investigación BIOARABA, Vitoria-Gasteiz, Ávala Spain; 6 Servicio de Neumología, Hospital Universitario Miguiel Servet, Zaragoza, Zaragoza Spain; 7 Instituto de Investigación Sanitaria de Aragón, Zaragoza, Spain; 8 Servicio de Neumología, Hospital Palma Planas, Palma, Balearic Islands Spain; 9 Servicio de Endocrinología, Hospital Universitari Son Espases, Palma, Balearic Islands Spain; 10 Servicio de Análisis Clínicos, Hospital Universitari Son Espases, Palma, Balearic Islands Spain; 11 Servicio de Pediatría, Hospital Universitari Son Espases, Palma, Balearic Islands Spain; 12 Servicio de Neumología, Hospital Universitario La Paz, IdiPaz, Madrid, Spain

**Keywords:** Gestational diabetes mellitus, Fetal outcomes, Cytokine; inflammation, Metanephrine, Normetanephrine, Obstructive sleep apnea

## Abstract

**Background:**

Although some evidence suggests an association between obstructive sleep apnea (OSA) and gestational diabetes mellitus (GDM), its consequences still remain largely unknown. We sought to determine whether OSA is associated with higher inflammation and sympathetic levels in GDM, and to relate them with insulin resistance and perinatal outcomes.

**Methods:**

OSA was identified by polysomnography and defined as an apnea–hypopnea index of ≥ 5 h^−1^. Plasma cytokines (TNF-α, IL-1β, IL-6, IL-8, IL-10), metanephrine, and normetanephrine were determined by immunoassays.

**Results:**

We included 17 patients with GDM and OSA and 34 without OSA. Women with GDM and OSA had higher normetanephrine concentrations [81 IQR (59–134) vs. 68 (51–81) pg/mL]. No differences in the inflammatory profile were found, while IL-1β was higher in patients with mean nocturnal oxyhemoglobin saturation ≤ 94%. We found positive correlations between increased sympathetic activation and IL-1β, with obstructive apneas, while time in REM showed an inverse relationship with IL-1β and metanephrine. Furthermore, IL-10 was inversely related with time in sleep stages 1–2, and with the arousal index, and it was positively related with time in slow-wave sleep. Significant correlations were also found between IL-1β and insulin resistance. There were no significant differences in neonatal characteristics; however, we found inverse relationships between IL-10 and birth weight (BW), and percentile of BW.

**Conclusions:**

OSA increased sympathetic activity, and IL-1β concentration was higher in patients with GDM with lower nocturnal oxygenation, all of which were related with obstructive events, and time in REM. Moreover, IL-1β was related with insulin resistance, and IL-10 inversely correlated with neonatal BW.

**Supplementary Information:**

The online version contains supplementary material available at 10.1186/s12890-022-01888-1.

## Background

Gestational diabetes mellitus (GDM), which is defined as glucose intolerance that is first detected during pregnancy, is a well-established risk factor for adverse maternal and infant health outcomes, including preeclampsia, caesarean delivery, fetal macrosomia, neonatal hypoglycemia, and fetal death. Numerous studies have identified risk factors for GDM, such as advance maternal age, previous GDM, obesity, and being member of some ethnic groups [1].

Obstructive sleep apnea (OSA) is characterized by repetitive episodes of total (apneas) or partial (hipoapneas) obstruction of the upper airway during sleep, and it is associated with oxygen desaturations and sleep disruption [2]. Some physiological changes, such as weight gain, hormonal variations, or modifications in the upper airway [3] increase prevalence of OSA during pregnancy. Some evidence suggests that OSA is associated with adverse pregnancy outcomes, such as gestational diabetes mellitus (GDM), preeclampsia, gestational hypertension, and with fetal related outcomes, preterm birth and neonatal low weight [4, 5]. Moreover, OSA and GDM share some risk factors, including obesity and increasing age. In addition, there is growing evidence that OSA is an independent risk factor for type 2 diabetes, and that it has an adverse influence on glycemic control [6].

Healthy pregnant women have higher plasma inflammation biomarkers compared to non-pregnant women [7]. Patients with GDM show additional inflammatory dysregulation [8]. Moreover, significant associations between maternal systemic inflammation and newborn birth weight have been found [8], although more studies are necessary with attention to confounding factors, such as age, obesity, and comorbidities.

The potential consequences of OSA in women with GDM have been poorly explained, and they still remain unclear. Among others, it has been suggested that frequent arousals and intermittent hypoxia could lead to an increase in inflammation, and in sympathetic activation, which could contribute to promote insulin resistance and GDM [3]. Nevertheless, most evidence of these multiple and complex mechanistic pathways linking GDM and OSA comes from non-pregnant populations [9–11], and it has yet to be demonstrated whether these mechanisms are also involved during pregnancy. A previous small study showed higher levels of interleukin (IL)-6 and IL-8, in 4 women with GDM and OSA compared to 21 women without OSA [12]. Moreover, a small study found decreased heart rate variability in pregnant women, a non-invasive marker of attenuation of parasympathetic activity [13].

The study aims were: (1) to compare the levels of inflammatory cytokines (tumor necrosis factor alpha (TNF-α), IL-1β, IL-6, IL-8, and IL-10), and plasmatic metanephrines concentrations in women with GDM with or without OSA, homogeneous in age and body mass index (BMI) in the third trimester; (2) to examine relations between the inflammatory/sympathetic profile and carbohydrate metabolism; and (3) to relate systemic inflammation/sympathetic profile with adverse maternal and fetal outcomes. As an exploratory objective we aimed to analyse the effect of nocturnal hypoxemia on systemic inflammation/metanephrines.

## Methods

### Subjects and study design

Women were recruited at three tertiary and university hospitals in Spain (Son Espases, Araba, and Miguel Servet). Inclusion criteria were: singleton pregnant women in the third trimester of pregnancy and GDM diagnosis, according to the fasting 3-h 100-g glucose tolerance testing [14]. Patients were excluded if they fulfilled at least one of the following exclusion criteria: (1) previous OSA diagnosis; (2) preeclampsia; (3) previous diagnosis of diabetes mellitus, lung, heart, or kidney diseases; (4) treatment with steroids; (5) unwillingness or inability to participate in the study; (6) imminent delivery due to maternal–fetal disease; or (7) any other concurrent severe medical condition that would contraindicate the patient´s participation in the study according to the investigators’ judgment [15].

Based on previous studies reporting plasma TNF-α levels of 10.4 +/− 2.1 pg/mL in the third trimester of pregnancy with GDM [16] and with an alpha risk of 0.05 and a beta risk in a two-way contrast, 34 healthy pregnant women and 17 pregnant women with OSA are required to detect a difference equal to or greater than 2.15 pg/mL (20.6% of mean value).

The study was approved by the Institutional Ethics Committee for all hospitals and all subjects gave their written informed consent.

### Clinical and sleep evaluation

Anthropometric, clinical, and sleep data, including Epworth Sleepiness Scale (ESS), were collected in all participants. The medical records of all enrolled women and their infants were reviewed after their discharge from the hospital, and the information regarding antepartum course and delivery complications was recorded. More detailed information is provided in supporting information.

### Polysomnography

Overnight-attended polysomnography (PSG) was performed in the sleep laboratory. Breathing was monitored using nasal cannulas, oronasal thermistors, and thoracoabdominal stain gauges. Simultaneously, oxyhemoglobin saturation (SaO_2_) was monitored with a pulse oximeter. Sleep was analyzed using the standard criteria for epochs of 30 s [17]. Apnea was defined as the absence of airflow (> 90% reduction) for at least 10 s while hypopnea was defined as a discernible airflow reduction (> 30% and < 90%) for at least 10 s with a ≥ 3% drop in SaO_2_ or final arousal. The events were considered obstructive in the presence of continued respiratory efforts. The apneas-hypopneas index (AHI) was established as the number of apneas and hypopneas per hour of sleep. OSA was defined as an AHI of ≥ 5 h^−1^ [18]. Rapid eye movement (REM) AHI was calculated as the number of apneas and hypopneas during REM sleep divided by total time in REM in hours. The mean SaO_2_ throughout the night, the minimum SaO_2_ (lowest values recorded during sleep), and the percentage of total time study spent with SaO_2_ < 90% (CT90%) were computed as indices of nocturnal SaO_2_.

### Blood sample collection and determinations

The morning after the PSG, anthropometric variables and blood samples were collected from all women in fasting conditions. Laboratory data included complete blood count (Cell-Dyn Sapphire Platform, Abbott Diagnostics), coagulation, kidney and liver function tests (Architect c16000 platform, Abbott Diagnostics, US). Insulin was determined by processing serum samples on the Cobas e-411 platform (Roche Diagnostics GmbH, Germany) with a reference range of 3–25 μUI/mL. The homeostatic model assessment of insulin resistance index (HOMA-IR) and quantitative insulin sensitivity check index (QUICKI) were calculated by the usual formula in those patients without insulin treatment [10].

The remaining blood sample was centrifuged at 2500 rpm for 10 min to isolate the plasma. The different plasma aliquots were stored at − 80 °C for further determinations.

The inflammatory cytokines were analyzed on plasma samples by multiplex technique using the Human High Sensitivity Cytokine Base Kit A (Magnetic Luminex^®^ Performance Assay, R&D Systems^®^, Inc.) following the indicated procedure. For each one of the cytokines analyzed, the detection limits and coefficients of variation were 0.29 pg/mL and 5.2% (TNF-α), 0.08 pg/mL and 5.3% (IL-1β), 0.14 pg/mL and 5.2% (IL-6), 0.04 pg/mL and 6.6% (IL-8), 0.21 pg/mL and 5.4% (IL-10).

Plasma metanephrine [detection range 15.1–3600 pg/mL, CV(%) 11.8] and normetanephrine concentrations [detection range 22.8–7200 pg/mL, CV(%) 9.32] hormones were measured with enzimoinmunoanalysis assay (ELISA) (Diasource Immunoassays SA).

### Statistical analysis

Data are presented as mean ± standard deviation, median + interquartile range (IQR) or percentage. Differences between groups were analyzed using Student’s t test or U Mann–Whitney test for continuous variables, and Fisher’s exact test (two-tailed) or chi-square test for categorical variables.

To analyze the effect of nocturnal hypoxia on systemic inflammation/metanephrines, we divided all included patients in two groups according to the mean SaO_2_ throughout the night: (a) mean SaO_2_ ≤ 94%; (b) mean SaO_2_ ≥ 95%.

Since the inflammatory biomarkers did not fit a normal distribution, their relationships with clinical variables were evaluated using the Spearman correlation. The statistical software SPSS v.26 (IBM) was used, and a two-sided *p* value less than 0.05 was considered significant.

## Results

### Subjects of study

To obtain the predetermined sample size for the experimental group, and to include 17 pregnant women classified as with OSA, we required assessing for eligibility 144 women with GDM. Fifty-five of them were not selected (45 refusals, 2 technical sleep study dropouts, 1 twin pregnancy, 4 deliveries before PSG, 1 change of address, and 2 because of language barriers), so we finally needed to perform PSG to 89 women. Among the remaining non-OSA group, we selected 34 women, who were homogeneous in age and body mass index (BMI) for comparative purposes.

Descriptive analysis of both groups for the main anthropometric and clinical variables and the laboratory findings are shown in Table 1. Hip circumference, plasmatic high-density lipoprotein (HDL), and hemoglobin concentration were significantly higher in women with OSA than in the non-OSA group. PSG characteristics, sleep symptoms, and specific anthropometric measurements of the two groups are illustrated in Table 2. As expected, there were significant differences in the AHI, REM AHI, and the obstructive apneas index (OAI). Besides, patients with OSA had worse nocturnal oxygenation indexes (minimum SaO_2_, mean SaO_2_ desaturation index, and CT90%SaO_2_) than the non-OSA group. No differences were found in daytime somnolence, snoring, episodes of subjective asphyxia, nocturia, morning headache, reported apneas, unrefreshing sleep, and reported sleep time between women with OSA and the control group (Table 2).

**Table 1:** General characteristics of women with GDM in OSA and non-OSA groups

Variables	Total (n = 51)	OSA (n = 17)	Non-OSA (n = 34)	*p* Value
Age (years)	37 (33–39)	38 (35–40)	35 (32–39)	0.120
Gestational age (weeks) at plasma sampling	36 (34–37)	36 (34–36.5)	36 (34.75–38)	0.551
BMI before pregnancy (kg/m^2^)	26.7 (23.3–30.1)	29.2 (24.9–32.4)	25.76 (23.2–29.1)	0.15
Obese before pregnancy n (%)	13 (25.5)	6 (35.3)	7 (20.6)	0.256
First pregnancy n (%)	26 (52)	8 (50)	18 (52.9)	0.846
*Pre-gestational smoker n (%)*				
No	27 (52.9)	7 (41.2)	20 (58.8)	0.393
Yes	15 (29.4)	7 (41.2)	8 (23.5)	
Former smoker	9 (17.6)	3 (17.6)	6 (17.6)	
Neck circumference (cm)	35.4 ± 3	35.1 ± 3.6	35.5 ± 2.8	0.634
Waist (cm)	110.2 ± 9.4	111 ± 11.4	109.8 ± 8.4	0.689
Hip circumference (cm)	115.7 ± 13.4	127.8 ± 18.3	112.1 ± 9.4	**0.008**
Glucose (mg/dL)	76.8 ± 8.9	74.5 ± 6.7	78 ± 9.6	0.186
Insulin (µUI/mL)^†^	11.8 (8.9–16.9)	18.2 (13.4–25.5)	13.4 (11.5–13.9)	0.137
HOMA-IR^†^	2.6 (1.7–3.2)	2.7 (1.8–4.9)	2.6 (1.2–2.8)	0.22
QUICKI^†^	0.33 (0.32–0.35)	0.33 (0.30–0.35)	0.33 (0.33–0.37)	0.202
Total cholesterol (mg/dL)	259 ± 57	258 ± 65	259 ± 54	0.952
Triglycerides (mg/dL)	221 (162–293)	216 (159–299)	232 (161–293)	0.865
HDL (mg/dL)	66 (58–75)	70 (60–82.5)	61 (50.5–72.5)	**0.029**
LDL (mg/dL)	144.9 ± 42.9	137.7 ± 35.3	148.5 ± 46	0.415
ALT (U/L)	16 (13–21)	17 (13.5–24)	15.5 (12–19.5)	0.275
GGT (U/L)	11 (7–16)	10 (7–15.5)	11 (7.8–16.5)	0.574
Hemoglobin (g/dL)	12.4 ± 0.14	12.8 ± 0.89	12.1 ± 1.04	**0.032**
Leucocytes (10^3^/uL)	8.5 ± 2.06	8.79 ± 2.18	8.36 ± 2.02	0.496
Platelets (10^3^/uL)	213 ± 8	195 ± 47	222 ± 60	0.112
Systolic BP (mmHg)	109 ± 12	111 ± 13	109 ± 12	0.990
Diastolic BP (mmHg)	68 ± 10	71 ± 11	66 ± 9	0.098

Bold indicates statistically significant differences between the groups

Descriptive analysis and comparison between groups. Continuous variables in mean ± SD if they follow normal distribution or in median (IQR) if they follow a non-parametric distribution. For categorical variables n (%). ^†^Limited to noninsulin users (n = 30). Abbreviations: *BMI* body mass index, *GDM* gestational diabetes mellitus, *HOMA-IR* homeostasis model assessment of insulin resistance, *HDL* high-density lipoprotein, *LDL* low-density lipoprotein, *ALT* alanine aminotransferase, *GGT* gamma glutamyl-transpeptidase, *BP* blood pressure, *g* grams, *kg* kilograms, *mg* milligrams, *m* meter, *cm* centimetre, *dL* deciliter, *U* units, *L* liter, *uL* microliter, *mmHg* millimeter of mercury

**Table 2:** Polysomnography data, sleep symptoms, and physical characteristics in OSA and non-OSA groups

Variables	Total	OSA (n = 17)	Non-OSA (n = 34)	*p* Value
Total sleep time, (min)	299 ± 61.1	283.3 ± 64.8	306.8 ± 58.6	0.198
N1 + N2 (%)	57.7 (31.5–69.3)	56 (19.2–68.8)	58 (47.2–70.8)	0.328
N3 (%)	26.5 (20.3–55.9)	31.3 (20.9–67.1)	24.3 (17.9–41.5)	0.328
REM sleep time (%)	11.41 ± 5.42	11.48 ± 4.34	11.37 ± 5.94	0.944
AHI (h^−1^)	0.9 (0.4–5.9)	7.6 (5.8–13.1)	0.5 (0.2–0.9)	**0.001**
OAI (h^−1^)	0 (0–0.3)	0.6 (0–1.3)	0 (0–0)	**0.001**
Arousal index (h^−1^)	13.1 (1.8–24.3)	16.9 (1.4–37.5)	12.3 (1.4–22)	0.349
REM AHI (h^−1^)	3.3 (0–8.6)	10.3 (6.4–23.5)	1.3 (0–3.7)	**0.001**
Mean SaO_2_ (%)	97.5 ± 1.4	96.6 ± 1.3	98 ± 1.1	**0.002**
Minimum SaO_2_ (%)	92 (89–94)	89 (86–91)	93 (91–94)	**0.002**
CT90%SaO_2_ (min)	0 (0–0.3)	0.2 (0–1)	0 (0–0)	**0.01**
Desaturation index (h^−1^)	0.2 (0–0.9)	0.9 (0–3.4)	0.2 (0–0.5)	**0.014**
Nocturia n (%)	42 (82.4)	15 (88.2)	27 (79.4)	0.359
Reported apneas, n (%)	5 (10.2)	3 (17.6)	2 (6.3)	0.210
Frequent gasping awakenings, n (%)	13 (25.5)	5 (29.4)	8 (23.5)	0.65
Morning headaches n (%)	9 (17.6)	2 (11.8)	7 (20.6)	0.436
Unrefreshing sleep n (%)	25 (50)	6 (56.2)	16 (47.1)	0.311
Snoring (always/frequent) n (%)	16 (31.4)	6 (35.3)	10 (29.4)	0.670
Reported sleep time, working days (h)	7.5 (7–8)	8 (6.5–9)	7 (6.9–8)	0.109
Reported sleep time, weekends (h)	8 (7–9)	8 (7–9)	8 (7–8)	0.156
Micrognatia n (%)	1 (2)	0 (0)	1 (2.9)	1.00
*Mallampati score n (%)*				
I–II	24 (47.1)	9 (52.9)	15 (44.1)	0.552
III–IV	27 (52.9)	8 (47.1)	19 (55.9)	
Epworth sleepiness scale	6.2 ± 3	7.1 ± 3	5.8 ± 2.9	0.154

Bold indicates statistically significant differences between the groups

Continuous variables in mean ± SD if they follow normal distribution or in median (IQR) if they follow a non-parametric distribution. For categorical variables n (%). Abbreviations: N1 + N2, time in sleep at stage 1 and 2; N3, time in sleep at stage 3; REM, rapid eye movement; AHI, apneas/hypopneas index; OAI, obstructive apneas index; SaO_2_, oxygen saturation; CT90%, sleep time with oxygen saturation < 90%; min, minutes; h^−1^, per hour; h, hours

With respect to non-apneic pregnant women, those with OSA did not show differences in the percentages of caesarean section (20% vs. 27.3%), induced deliveries (33.3% vs. 28.6%), instrumented deliveries (25% vs. 28.6%), and vaginal candidiasis (9.1% vs. 8.7%).

### OSA and inflammatory/sympathetic profile

**Fig. 1:**
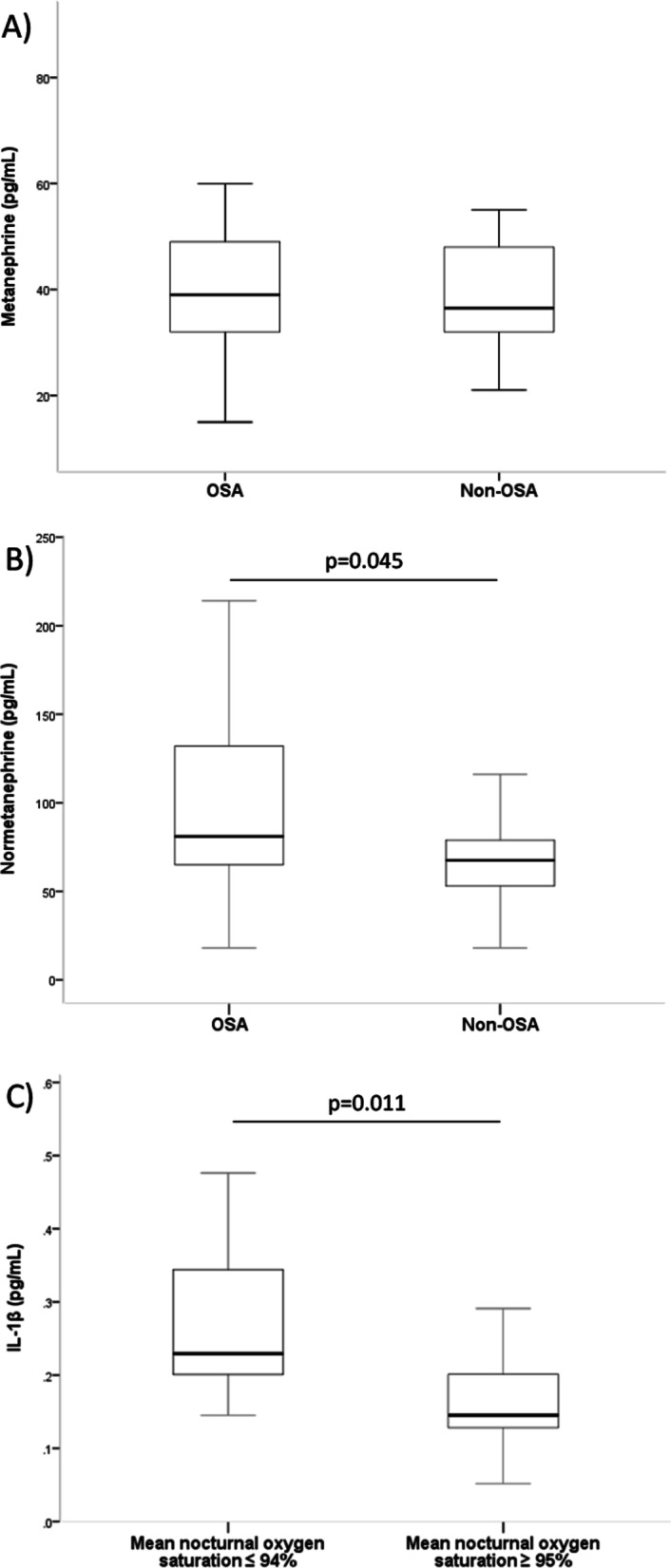
Metanephrine, normetanephrine, and IL-1β concentrations in women with GDM. **A** metanephrine, **B** normetanephrine, **C** IL-1β. Boxplots represent medians and interquartile ranges. Abbreviations: *IL* interleukin, *OSA* obstructive sleep apnea

Patients with OSA had significant higher levels of normetanephrine compared to pregnant women without OSA [81 IQR (59–134) vs. 68 (51–81) pg/mL, *p* = 0.045] (Fig. 1). However, we did not find differences in plasmatic levels of metanephrine, TNF-α, IL-1β, IL-6, IL-8, and IL-10 (Table 3).

**Table 3:** Sympathetic and inflammatory profiles in women with GDM in OSA and non-OSA groups

Variables	OSA (n = 17)	Non-OSA (n = 34)	*p* Value
Metanephrine (pg/mL)	39 (31–52)	37 (32–48)	0.696
Normetanephrine (pg/mL)	81 (59–134)	68 (51–81)	**0.045**
TNF-α (pg/mL)	6.91 (5.43–8.26)	7.51 (6.45–10.49)	0.204
IL-1β (pg/mL)	0.16 (0.12–0.34)	0.15 (0.13–0.20)	0.253
IL-6 (pg/mL)	1.32 (0.97–1.67)	1.13 (0.84–1.37)	0.204
IL-8 (pg/mL)	2.08 (1.25–2.93)	1.94 (1.37–3.04)	0.905
IL-10 (pg/mL)	0.51 (0.32–1.05)	0.51 (0.37–0.97)	0.976

Bold indicates statistically significant differences between the groups

Variables in median (IQR). Abbreviations: *GDM* gestational diabetes mellitus, *IL* interleukin, *pg* picograms, *mL* millilitre, *TNF-α* tumor necrosis factor alpha

We identified 7 GDM patients with mean nocturnal SaO_2_ ≤ 94% (5 of them presented AHI ≥ 5 h^−1^), and the remaining 44 women had SaO_2_ ≥ 95%. We found higher IL-1β levels in patients with mean nocturnal SaO_2_ ≤ 94% compared to those women with SaO_2_ ≥ 95% (Fig. 1C). In addition, there were higher levels of normetanephrine in patients with nocturnal SaO_2_ ≤ 94%, but they did not reach statistical significance [124 IQR (45–154) vs. 70 (54–88) pg/mL, *p* = 0.087]. The remaining inflammatory and sympathetic biomarkers were not different according to nocturnal oxygen (Additional file 1: Table S1).

### Relation between the inflammatory/sympathetic profile and sleep characteristics

In the set of women with GDM, normetanephrine level was mildly-moderately related to OAI, mean duration of obstructive apneas, AHI, and supine AHI, while metanephrine was only moderately related to time in REM stage. Some relationships between sleep characteristics and inflammatory biomarkers were also identified. Thus, IL-1β levels were moderately related with time in REM and mildly-moderately with OAI, as well as maximum and mean length of obstructive apneas (Table 4). Furthermore, significant moderate positive relationships were also found between IL-6 and arousal index (rho = 0.48, *p* = 0.001), while IL-10 was inversely and moderately related with time in sleep stages 1 and 2 (N1 + N2) (rho = -0.36, *p* = 0.01) and arousal index (rho =  − 0.33, *p* = 0.021), and it was positively related with time in sleep stage 3 (N3) (rho = 0.36, *p* = 0.009).

**Table 4:** Relationship between metanephrines and IL-1β plasmatic concentrations, and sleep parameters in women with GDM

	Metanephrine			Normetanephrine			IL-1β		
	Correlation coefficient		*p* Value	Correlation coefficient		*p* Value	Correlation coefficient		*p* Value
	rho	95%CI		rho	95%CI		rho	95%CI	
REM sleep time, %	− 0.334	− 0.558 to − 0.064	0.017	–	–	–	− 0.328	− 0.554 to − 0.057	0.019
Obstructive apnea index, h^−1^	–	–	–	0.329	0.058–0.555	0.018	0.289	0.014–0.523	0.040
Number of obstructive apneas	–	–	–	0.319	0.047–0.547	0.022	0.280	0.004–0.516	0.047
Mean obstructive apneas length, s	–	–	–	0.287	0.012–0.522	0.041	0.286	0.011–0.521	0.042
Maximum obstructive apneas length, s	–	–	–	–	–	–	0.319	0.047–0.547	0.026
Apnea-hypopneas index, h^−1^	–	–	–	0.3	0.059–0.555	0.033	–	–	–
Number of apnea-hypopneas	–	–	–	0.294	0.02–0.527	0.036	–	–	–
Apnea-hypopneas supine index, h^−1^	–	–	–	0.329	0.058–0.555	0.044	–	–	–

Abbreviations: *GDM* gestational diabetes mellitus, *IL* interleukin

### Relation between the inflammatory/sympathetic profile and carbohydrate metabolism

**Fig. 2:**
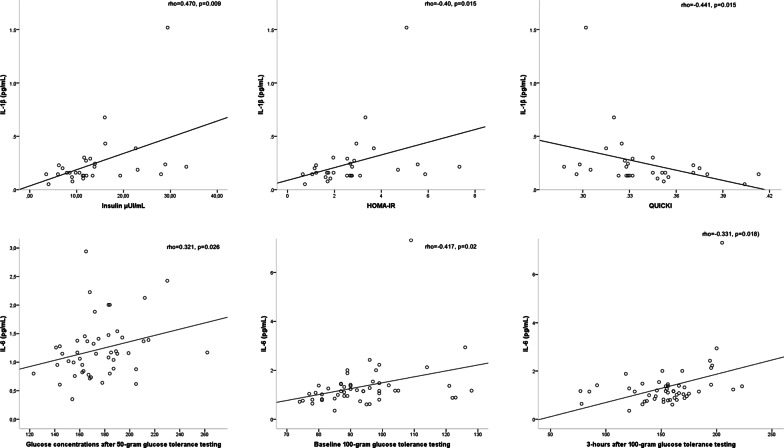
Relationships between the inflammatory profile, carbohydrate metabolism and insulin resistance in women with GDM. Boxplots represent medians and interquartile ranges. Abbreviations: *IL* interleukin

In 30 patients that were not treated with insulin, higher levels of IL-1β were associated with higher insulin concentration as well as with more resistance and less sensitivity to insulin, which was assessed by the HOMA-IR and QUICKI indices, respectively. IL-6 was positively and moderately related with glucose concentrations after 50 g glucose tolerance testing, and baseline (rho = 0.417, *p* = 0.002), and three hours after 100 g glucose tolerance testing (Fig. 2). In contrast, IL-10 showed inverse and moderate relation with glucose concentration 3 h after 100-g glucose tolerance testing (rho = -0.331, *p* = 0.018).

In regard to nocturnal oxygenation, we found higher insulin concentration in patients with a mean nocturnal SaO_2_ ≤ 94% compared to pregnant women with SaO_2_ ≥ 95% [22.6 IQR (12.7–25.5) vs. 11.4 (8.2–15) µUI/mL, *p* = 0.049]. Additionally, higher levels of HOMA-IR and lower levels of QUICKI were shown in patients with SaO_2_ ≤ 94%, but they did not reach statistical significance (both *p* = 0.055).

### Relation between the inflammatory/sympathetic profile and neonatal characteristics

One patient was excluded in the analysis of the neonatal outcomes as she had moderate OSA and she was on continuous positive airway pressure after PSG. Neonatal outcomes are presented in Additional file 1: Table S2. One baby had preterm birth and one pregnant woman had preeclampsia; both of them presented AHI ≥ 5 h^−1^. We found no significant differences in the neonatal characteristics based on the presence of OSA in pregnant women. However, IL-10 levels were inversely and mildly correlated with birth weight (rho =  − 0.283, *p* = 0.049) and moderately correlated with percentile of birth weight (rho =  − 0.332, *p* = 0.023).

## Discussion

Our study provides some interesting findings. In the first place, women with GDM and OSA had significant higher plasmatic normetanephrine concentration compared to women without OSA. In contrast, there were no differences in metanephrine, TNF-α, IL-1β, IL-8, and IL-10, while IL-1β levels were higher in patients with a mean SaO_2_ ≤ 94% during sleep. Secondly, there were significant correlations between increased sympathetic activation and IL-1β, with respiratory obstructive sleep events and time in REM during sleep. In the third place, systemic inflammation was related with glucose metabolism and insulin resistance. Finally, IL-10 during the third trimester was inversely correlated with neonatal birth weight.

### Sleep symptoms in OSA and non-OSA

Previous studies did not find differences in subjective sleep time or daytime sleepiness when comparing OSA and non-OSA pregnant women with GDM [15, 19]. Also, ESS shows low predictive value of OSA during pregnancy [20]. Similarly, there were no differences in reported sleep time or ESS between our study groups. Our study aim was to compare inflammatory/sympathetic biomarkers in women with GDM with and without OSA, and the sample size of the present study may be not sufficiently powered to find differences in the sleep symptoms. As other studies, we did not find differences in subjective self-reported OSA symptoms among OSA and control subjects (Table 2) [15, 19].

### Inflammatory/sympathetic profile and OSA

OSA prevalence increases during pregnancy [3], and it has been associated with GDM [4, 19]. The mechanisms underlying this association are still unknown and most likely multifactorial. It has been suggested that persistent apneas/hypopneas, together with arousals, results in intermittent hypoxia, sleep fragmentation, and reductions in total sleep duration and time in REM, which could trigger physiologic consequences that include, among others, sympathetic activity and inflammation that could lead to insulin resistance and glucose intolerance [21]. However, currently, there is no clear evidence of the effect of OSA on these specific mechanisms in GDM.

Plasma noradrenaline concentrations during late-night period are higher during pregnancy compared with non-pregnant women [22]. Patients with GDM may further increase sympathetic activity, although the results are inconsistent [23].

Moreover, numerous studies have shown elevated levels of catecholamines among non-pregnant patients with OSA, which are attenuated by treatment [24]. In contrast, there is only one small study including 64 healthy pregnant women that studied indirectly its influence. In this study, the authors found a shift toward increased sympathetic tone based on changes in heart rate variability in only three women with associated deep nocturnal oxygen desaturation [13]. In the present study, higher normetanephrine levels were found in OSA patients. Besides, we showed significant correlations between normetanephrine and obstructive apneas and hypopneas (Table 4), while metanephrine was inversely related to percentage of time in REM. As far as we know, this is the first description on the influence of repetitive nocturnal obstructive apneas events in the overall sympathetic activation in GDM.

Low grade inflammation is a common event in the third trimester in normal pregnancies, and it is necessary for the correct evolution throughout gestation [7]. Both proinflammatory cytokines concentrations, such as IL-1β, IL-6 and IL-8 [7, 25], and anti-inflammatories, such as IL-10 [26], increase during healthy pregnancy. Moreover, GDM has been related to up-regulation of inflammatory biomarkers [8, 27]. The prevailing evidence also suggests that OSA increases the concentration of proinflammatory cytokines in non-pregnant populations [9–11]. However, there is only a previous study showing higher levels of IL-6, and IL-8 in a small group of women with GDM and OSA compared to women without OSA [12]. Besides, the latter cytokines, as well as TNF-α, were significantly related with AHI. Nevertheless, these findings should be interpreted cautiously since no differences were found in BMI, and only four women with OSA with mean BMI of 36.4 kg/m^2^ were included. Moreover, OSA status was diagnosed at home with a type III portable device, which has not been validated in pregnant women. On the other hand, patients were classified according to PSG results in the present study, which included only 25% of patients with obesity; although we found no significant differences in the inflammatory markers comparing OSA with non-OSA, we did find correlations between IL-1β and obstructive apneas, and time in REM. Furthermore, IL-10 was inversely related with sleep fragmentation and positively related with amount of slow wave sleep time. Possible explanations for this finding are, among others, that inflammation markers are already increased in normal pregnancy and in GDM [8], which might have masked any potential further effect of OSA on systemic inflammation. Moreover, the sample size may be underpowered to find significant differences. Finally, most women of the present study presented mild OSA. Adaptive mechanisms may exist in mild stages of OSA in GDM, which may be overcome when patients are exposed to a greater degree of hypoxia and greater sleep fragmentation above an hypothetical threshold that is currently unknown. In fact, repetitive cycles of intermittent hypoxia are characteristic features of OSA that promote IL-1β production [28]. Interestingly, our results show higher IL-1β levels in those women with mean SaO_2_ ≤ 94% during sleep. This finding suggests that nocturnal hypoxia may result in up-regulation of IL-1β in patients with GDM.

### Relation between the inflammatory/sympathetic profile, carbohydrate metabolism, and insulin resistance

Diabetes is characterized by the presence of increased proinflammatory cytokines, being IL-1β one of the main inflammatory markers associated in the reduction of insulin signaling leading to insulin resistance in GDM [29]. In fact, IL-1β blocking therapies have been reported to improve glycemia in patients with type 2 diabetes [30]. Besides, increased sympathetic activity has a negative impact on glucose metabolism [31]. We have shown that IL-1β and insulin were higher in those women with lower mean SaO_2_ and that there were significant relationships between IL-1β, insulin concentration, HOMA-IR, and QUICKI. In addition, inverse relationships between IL-1β, and metanephrine, with time in REM during sleep have been found. Hypoxia markers have been positively correlated with fasting glucose levels together with beta cell dysfunction [32, 33] in previous studies. Moreover, REM decreases in the third trimester of gestation, which is further reduced both in pregnant and non-pregnant population because of OSA [34, 35]. Therefore, it could be speculated that apneas occurring during pregnancy, when combined with the effects of insufficient REM sleep and nocturnal hypoxia, could potentially promote insulin resistance mediated through increments in IL-1β and in plasma catecholamine concentrations. Nonetheless, few studies with very small samples and some confounding variables have examined whether nocturnal hypoxia increases the risk of IR; therefore, future studies are required to further explore the role of OSA, hypoxia, REM and these biomarkers during pregnancy, as well as their influence in GDM.

### Inflammatory profile and pregnancy and neonatal outcomes

Fetal macrosomia is a common adverse infant outcome of GDM if it is not treated in time, which increases the risk of adverse consequences to both the mother and the offspring, including shoulder dystocia, clavicle fractures, brachial plexus injury, neonatal intensive care unit admissions, cesarean delivery, and postpartum hemorrhage [36]. Our results showed that a higher presence of sleep fragmentation and a lower presence of less slow wave sleep resulted in a lower the IL-10 concentration. In addition to this, we found significant inverse correlations of the newborn's birthweight with IL-10. All of which might suggest that IL-10, as a counter-regulatory anti-inflammatory mechanism, could influence this essential clinical outcome. Nevertheless, whether proinflammatory/anti-inflammatory cytokine imbalance in women with GDM and OSA will result in increased birthweight is still to be determined in future studies.

### Strengths and limitations

This study has some strengths, such as originality, a multicenter and prospective design, and careful selection of GDM patients with and without OSA, who were homogeneous in age and BMI, and rigorous sleep and clinical phenotyping, including attended PSG.

Furthermore, researchers were blinded to OSA status. Yet, as in any study, there are some limitations that deserve to be commented on. First, the sample size was small for certain comparisons, because it was challenging to include GDM patients with newly diagnosed OSA but otherwise healthy (prevalence is around 15%). Second, the sample size calculation was based on detection of TNF-α level differences, as it was the most previously studied biomarker both in OSA and GDM. Despite not finding differences in the cytokine’s levels (including TNF-α), the number of individuals included in the study was sufficiently powered to observe significant differences in plasmatic normetanephrine concentration according to OSA status, as well as to find significant correlations between normetanephrine and obstructive apneas and hypopneas. Third, our results could indicate that nocturnal hypoxia may result in up-regulation of IL-1β in GDM patients. Nevertheless, this finding should be interpreted cautiously as it was an exploratory objective, and the sample size of women with mean nocturnal SaO_2_ ≤ 94% was very small. What is more, further studies are needed to better verify the influence of nocturnal hypoxemia on systemic inflammation to a greater extent, given that women of the present study had very mild impact of OSA on nocturnal hypoxemia indexes, which limited any other further analysis. Fourth, since our study included mainly Caucasian women, our results may not be directly appropriate in other ethnic backgrounds.

## Conclusions

To conclude, we have found that women with GDM and OSA had a higher sympathetic tone, which was assessed by normetanephrine plasmatic levels, compared to patients with GDM without OSA. In contrast, there were no differences in inflammatory profile, although IL-1β levels were higher in patients with lower nocturnal oxygenation. Both IL-1β and sympathetic activity were related to respiratory obstructive sleep events and time in REM during sleep. Moreover, IL-1β in GDM was related with glucose metabolism and insulin resistance, and IL-10 inversely correlated with neonatal birth weight. To our knowledge, this is the first description on the influence of OSA and nocturnal hypoxia in systemic inflammation and plasmatic metanephrines in GDM. These findings suggest there could be at least two underlying mechanisms promoting insulin resistance, although OSA influence on systemic inflammation and sympathetic activity in GDM might be more complex than its effect on AHI would implicate. Further, and larger investigations, including healthy pregnant women, and controlling for confounding factors are needed to better clarify and characterize the potential complex interrelationships between OSA, systemic inflammation, and sympathetic activity in GDM and the impact in perinatal outcomes.

## Supplementary Information

**Additional file 1.** Clinical and sleep evaluation and supporting **Table 1**. Details about clinical and sleep evaluation.

## Data Availability

In accordance with Spanish laws, the research team cannot share the full database that was used for the current paper. Moreover, data contain potentially identifying or sensitive patient information. However, other researchers who meet the criteria for access to confidential data may request to gain access to the minimal data set underlying the results under request at the Research Ethics Committee (contact via https://www.caib.es/sites/comiteetic/es/portada44578/?campa=yes), (e-mail address: ceic_ib@caib.es).

## Abbreviations

OSA: Obstructive sleep apnea
GDM: Gestational diabetes mellitus
TNF-α: Tumour necrosis factor alpha
IL: Interleukin
BW: Body weight
BMI: Body mass index
ESS: Epworth sleepiness scale
PSG: Polysomnography
SaO_2_: Oxyhemoglobin saturation
AHI: Apnoea hypopnea index
REM: Rapid eye movement
CT90%: Percentage of total time study spent with SaO_2_ < 90%
QUICKI: Quantitative insulin sensitivity check index
HOMA-IR: Homeostatic model assessment of insulin resistance index
IQR: Median interquartile range
HDL: High density lipoprotein
OAI: Obstructive apnea index

## References

[1] ACOG Practice Bulletin No (2018). 190: gestational diabetes mellitus. Obstet Gynecol.

[2] Mediano O, González Mangado N, Montserrat JM, Alonso-Álvarez ML, Almendros I, Alonso-Fernández A (2021). Documento internacional de consenso sobre apnea obstructiva del sueño. Arch Bronconeumol.

[3] Pamidi S, Kimoff RJ (2018). Maternal sleep-disordered breathing. Chest.

[4] Facco FL, Parker CB, Reddy UM, Silver RM, Koch MA, Louis JM (2017). Association between sleep-disordered breathing and hypertensive disorders of pregnancy and gestational diabetes mellitus. Obstet Gynecol.

[5] Warland J, Dorrian J, Morrison JL, O’Brien LM (2018). Maternal sleep during pregnancy and poor fetal outcomes: a scoping review of the literature with meta-analysis. Sleep Med Rev.

[6] Martínez-Ceron E, Fernández-Navarro I, Garcia-Rio F (2016). Effects of continuous positive airway pressure treatment on glucose metabolism in patients with obstructive sleep apnea. Sleep Med Rev.

[7] Nadeau-Vallée M, Obari D, Palacios J, Brien MÈ, Duval C, Chemtob S (2016). Sterile inflammation and pregnancy complications: a review. Reproduction.

[8] Atègbo J-M, Grissa O, Yessoufou A, Hichami A, Dramane KL, Moutairou K (2006). Modulation of adipokines and cytokines in gestational diabetes and macrosomia. J Clin Endocrinol Metab.

[9] Ming H, Tian A, Liu B, Hu Y, Liu C, Chen R (2018). Inflammatory cytokines tumor necrosis factor-α, interleukin-8 and sleep monitoring in patients with obstructive sleep apnea syndrome. Exp Ther Med.

[10] Martínez-Cerón E, Barquiel B, Bezos A-M, Casitas R, Galera R, García-Benito C (2016). Effect of continuous positive airway pressure on glycemic control in patients with obstructive sleep apnea and type 2 diabetes. A randomized clinical trial. Am J Respir Crit Care Med.

[11] Cao Y, Song Y, Ning P, Zhang L, Wu S, Quan J (2020). Association between tumor necrosis factor alpha and obstructive sleep apnea in adults: a meta-analysis update. BMC Pulm Med.

[12] Bublitz MH, Carpenter M, Amin S, Okun ML, Millman R, De La Monte SM (2018). The role of inflammation in the association between gestational diabetes and obstructive sleep apnea: a pilot study. Obstet Med.

[13] Watanabe M, Shinohara H, Kodama H (2015). Impact of overnight oximetry findings on cardiac autonomic modulation in women during second trimester of uncomplicated pregnancy. J Obstet Gynaecol Res.

[14] Ricart W, López J, Mozas J, Pericot A, Sancho MA, González N (2005). Potential impact of American Diabetes Association (2000) criteria for diagnosis of gestational diabetes mellitus in Spain. Diabetologia.

[15] Alonso-Fernández A, Moncadas MC, De Larrinaga AÁR, Barón AS, Marcet MC, Rodríguez PR, Gómez AVG, Carrero MPG, Martínez CP, Marín JPC (2021). Impact of obstructive sleep apnea on gestational diabetes mellitus. Arch Bronconeumol.

[16] Saucedo R, Zarate A, Basurto L, Hernandez M, Puello E, Galvan R (2011). Relationship between circulating adipokines and insulin resistance during pregnancy and postpartum in women with gestational diabetes. Arch Med Res.

[17] Conrad Iber, Sonia Ancoli-Israel ALCJ e SFQ. The AASM manual for the scoring of sleep and associated events: rules, terminology and technical specifications. Westchester, IL: The American Academy of Sleep Medicine. 2007, p. 59.

[18] Berry RB, Budhiraja R, Gottlieb DJ, Gozal D, Iber C, Kapur VK (2012). Rules for scoring respiratory events in sleep: update of the 2007 AASM manual for the scoring of sleep and associated events. Deliberations of the sleep apnea definitions task force of the American Academy of Sleep Medicine. J Clin sleep Med JCSM Off Publ Am Acad Sleep Med.

[19] Izci Balserak B, Pien GW, Prasad B, Mastrogiannis D, Park C, Quinn LT (2020). Obstructive sleep apnea is associated with newly diagnosed gestational diabetes mellitus. Ann Am Thorac Soc.

[20] Izci-Balserak B, Zhu B, Gurubhagavatula I, Keenan BT, Pien GW (2019). A screening algorithm for obstructive sleep apnea in pregnancy. Ann Am Thorac Soc.

[21] Abdul Jafar NKF, Eng DZ, Cai S (2019). Sleep in pregnancy and maternal hyperglycemia: a narrative review. Curr Diab Rep.

[22] Pöyhönen-Alho M, Viitasalo M, Nicholls MG, Lindström B-M, Väänänen H, Kaaja R (2010). Imbalance of the autonomic nervous system at night in women with gestational diabetes. Diabet Med.

[23] Feng Y, Feng Q, Qu H, Song X, Hu J, Xu X (2020). Stress adaptation is associated with insulin resistance in women with gestational diabetes mellitus. Nutr Diabetes.

[24] Kohler M, Stoewhas A-C, Ayers L, Senn O, Bloch KE, Russi EW (2011). Effects of continuous positive airway pressure therapy withdrawal in patients with obstructive sleep apnea: a randomized controlled trial. Am J Respir Crit Care Med.

[25] Orsi NM, Tribe RM (2008). Cytokine networks and the regulation of uterine function in pregnancy and parturition. J Neuroendocrinol.

[26] Okun ML, Coussons-Read ME (2007). Sleep disruption during pregnancy: how does it influence serum cytokines?. J Reprod Immunol.

[27] Lekva T, Norwitz ER, Aukrust P, Ueland T (2016). Impact of systemic inflammation on the progression of gestational diabetes mellitus. Curr Diab Rep.

[28] Ala Y, Palluy O, Favero J, Bonne C, Modat G, Dornand J (1992). Hypoxia/reoxygenation stimulates endothelial cells to promote interleukin-1 and interleukin-6 production. Effects of free radical scavengers. Agents Actions.

[29] Lappas M (2014). Activation of inflammasomes in adipose tissue of women with gestational diabetes. Mol Cell Endocrinol.

[30] Cavelti-Weder C, Babians-Brunner A, Keller C, Stahel MA, Kurz-Levin M, Zayed H (2012). Effects of gevokizumab on glycemia and inflammatory markers in type 2 diabetes. Diabetes Care.

[31] Briançon-Marjollet A, Weiszenstein M, Henri M, Thomas A, Godin-Ribuot D, Polak J (2015). The impact of sleep disorders on glucose metabolism: endocrine and molecular mechanisms. Diabetol Metab Syndr.

[32] Sökücü SN, Karasulu L, Dalar L, Özdemir C, Seyhan EC, Aydin Ş (2013). Efecto de la hipoxia sobre el metabolismo de la glucosa en pacientes no diabéticos con síndrome de apnea obstructiva del sueño. Arch Bronconeumol.

[33] Wanitcharoenkul E, Chirakalwasan N, Amnakkittikul S, Charoensri S, Saetung S, Chanprasertyothin S (2017). Obstructive sleep apnea and diet-controlled gestational diabetes. Sleep Med.

[34] Redline S, Kirchner HL, Quan SF, Gottlieb DJ, Kapur V, Newman A (2004). The effects of age, sex, ethnicity, and sleep-disordered breathing on sleep architecture. Arch Intern Med.

[35] Garbazza C, Hackethal S, Riccardi S, Cajochen C, Cicolin A, D’Agostino A (2020). Polysomnographic features of pregnancy: a systematic review. Sleep Med Rev.

[36] Metzger BE, Lowe LP, Dyer AR, Trimble ER, Chaovarindr U, Coustan DR (2008). Hyperglycemia and adverse pregnancy outcomes. N Engl J Med.

