# Supplementary material for: Systemic inflammation and sympathetic activation in gestational diabetes mellitus with obstructive sleep apnea
Source: BMC Pulm Med. 2022 Mar 18;22:94. doi: 10.1186/s12890-022-01888-1 (PMC8933971; doi:10.1186/s12890-022-01888-1)
Supplement: Supplementary file 1 — Additional file 1. Clinical and sleep evaluation and supporting Table 1. Details about clinical and sleep evaluation. [file 12890_2022_1888_MOESM1_ESM.docx]

**METHODS**

Clinical and sleep evaluation

Each patient had their anthropometric, clinical, and sleep data collected by means of questionnaires and direct measurements that included: age, BMI, and co-morbidities. Neck, waist, and hip circumferences were measured at the levels of the cricothyroid membrane, at the equidistant point between the iliac crest and the lowest rib, and at the point of greater trochanter, respectively. Office blood pressure was measured by a random-zero sphygmomanometer with an appropriate-sized cuff, as the individual remained seated for at least 5 minutes. Recorded values were the mean of three readings.

Episodes of subjective gasping, snoring, bed partner reported sleep breathing pauses during sleep, nocturia, morning headache, morning tiredness and sleepiness while driving were collected in four degrees of intensity (never, sometimes, frequently, and always) based on the previous four weeks. Moreover, subjective nocturnal sleep time on working days and weekends along with daytime sleepiness using the Epworth sleepiness scale (ESS)^1^ based on the previous four weeks were ascertained. Daytime sleepiness was defined as an ESS≥11.

All participants received obstetrical care by their physicians, who were unaware of the sleep study results, and no modification of obstetrical care was proposed for study participants.

Maternal and neonatal outcomes were collected postpartum from the medical records. Data included: age, race, obstetric history, gestational age at enrolment, pre-pregnancy weight and BMI, total weight gain during pregnancy, parity, co-morbidities, pregnancy-related complications (preeclampsia, proteinuria, preterm delivery, vaginal candidiasis, miscarriages), mode of delivery (vaginal, caesarean section, instrumented delivery, spontaneous or induced labor), term at delivery, birth weight,^2^ birth length, head circumference, acid-base status, and 1 and 5-min Apgar scores.

Preeclampsia was defined as; new onset hypertension (systolic blood pressure≥140 mmHg or diastolic blood pressure≥90 mmHg) starting at or after 20 weeks of pregnancy in a woman with previously normal blood pressure with documented proteinuria. Proteinuria was defined as total protein excretion of 300 mg or more in a 24-hour urine sample. Preterm delivery was defined as delivery before 37 weeks and 0 days of gestation by best obstetrical estimate.

**REFERENCES**

1. E. Chiner, J. M. Arriero, J. Signes-Costa, J. Marco, and I. Fuentes. Validation of the Spanish versión of the Epworth Sieepiness Scale in patients with sieep apnea syndrome. *Arch. Bronconeumol.* 1999;35(9):422–427.
2. A Carrascosa Lezcano, J M Fernández García, C Fernández Ramos et al. Spanish cross-sectional growth study 2008. Part II. Height, weight and body mass index values from birth to adulthood. *An Pediatr (Barc)*. 2008;68(6):552-69.

**Supporting table 1. Relations between inflammatory/sympathetic profiles according to nocturnal oxygen saturation in pregnant women with GDM.**

| **Variables** | **SaO_2_≤94% (n=7)** | **SaO_2_≥95%**  **(n=44)** | **P value** |
| --- | --- | --- | --- |
| **TNF-α (pg/mL)** | 7.39 (6.3-11.69) | 7.39 (5.89-9.15) | 0.511 |
| **IL-1β (pg/mL)** | 0.23 (0.19-0.39) | 0.15 (0.13-0.2) | 0.011 |
| **IL-6 (pg/mL)** | 1.15 (0.78-1.32) | 1.16 (0.88-1.43) | 0.613 |
| **IL-8 (pg/mL)** | 1.72 (0.97-2.48) | 2.05 (1.38-3.15) | 0.427 |
| **IL-10 (pg/mL)** | 0.51 (0.34-1.02) | 0.52 (0.37-0.99) | 0.816 |
| **Metanephrine (pg/mL)** | 34 (32-58) | 37 (31-49) | 0.968 |
| **Normetanephrine (pg/mL)** | 124 (45-154) | 70 (54-88) | 0.087 |

Continuous variables in median (IQR).. Abbreviations: GDM, gestational diabetes mellitus; IL, interleukin; mL, milliliter; pg, picograms; SaO_2_, oxyhemoglobin saturation; TNF-α, tumor necrosis factor alpha.

**Supporting table 2: Neonatal characteristics in OSA and non-OSA groups.**

| **Variables** | | **OSA (n=16)** | **Non-OSA (n=34)** | **P value** |
| --- | --- | --- | --- | --- |
| **Birth weight (Kg)** | | 3.09±0.4 | 3.24±0.36 | 0.137 |
| **Birth weight (percentile)** | | 32.5 (22.8-64) | 52 (29-79) | 0.132 |
| **Birth length (cm)** | | 49.2±2 | 50.4±1.7 | 0.067 |
| **Head circumference (cm)** | | 34.5 (32.5-36) | 34.5 (34-35) | 0.559 |
| **Umbilical cord pH** | | 7.33 (7.29-7.36) | 7.28 (7.25-7.32) | 0.06 |
| **Apgar 1^st^ min** | | 9 (9-9) | 9 (9-9) | 0.753 |
| **Apgar 5^th^ min** | | 10 (10-10) | 10 (10-10) | 0.98 |
| **Gender**  **n (%)** | **M** | 8 (50) | 19 (57.6) | 0.617 |
|  | **F** | 8 (50) | 14 (42.4) |  |
| **Neonatal gestational age (weeks)** | | 40 (38-41) | 40 (39-40.8) | 0.874 |

Continuous variables in mean ± sd if they follow normal distribution or in median (IQR) if they follow a non-parametric distribution.
